# Upadacitinib dose adjustment in moderate‐to‐severe atopic dermatitis: insights from a multicenter real‐world study

**DOI:** 10.1111/ddg.15908

**Published:** 2025-08-18

**Authors:** Flavia Manzo Margiotta, Valentina Dini, Laura Calabrese, Laura Lazzeri, Sofia Lo Conte, Manfredi Magliulo, Giulio Montesi, Leonardo Pescitelli, Nicola Milanesi, Carlo Mazzatenta, Emiliano Antiga, Pietro Rubegni, Massimo Gola, Marco Romanelli, Alessandra Cartocci

**Affiliations:** ^1^ Department of Dermatology University of Pisa Pisa Italy; ^2^ Health Science Interdisciplinary Center Sant'Anna School of Advanced Studies Pisa Italy; ^3^ Dermatology Unit Department of Medical Sciences Surgery and Neurosciences University of Siena Siena Italy; ^4^ Unit of Diagnostic and Therapeutic Neuroradiology Department of Neurology and Human Movement Sciences Azienda Ospedaliero Universitaria Siena Italy; ^5^ Allergological and Pediatric Dermatology Unit Department of Health Sciences University of Florence Florence Italy; ^6^ Unit of Dermatology San Giuseppe Hospital Empoli Florence Italy; ^7^ Department of Dermatology San Jacopo Hospital Pistoia Italy; ^8^ Unit of Dermatology Azienda USL Toscana Nord Ovest Lucca Italy; ^9^ Department of Health Sciences Section of Dermatology University of Florence Florence Italy

**Keywords:** atopic dermatitis, dose, efficacy, JAK inhibitors, Upadacitinib

Dear Editors,

In recent years, biologic therapies and small‐molecule agents, particularly Janus kinase (JAK) inhibitors, have emerged as transformative treatment options for patients with moderate‐to‐severe atopic dermatitis (AD).^1^ These therapies offer rapid and targeted relief for a disease characterized by chronic inflammation, severe pruritus, and significant impairment in quality of life. Among the JAK inhibitors, upadacitinib – an oral selective JAK1 inhibitor – has demonstrated significant efficacy in reducing disease severity, itch intensity, and sleep disturbances across various clinical trials.^2^ Both the 15 mg and 30 mg daily dose regimens have been associated with marked improvements, providing clinicians with the flexibility to tailor treatment based on disease severity and patient‐specific factors. This dose flexibility is particularly important in real‐world clinical practice, where patient profiles often diverge from those of individuals enrolled in randomized controlled trials. The ability to adjust dosages based on patient response and tolerance enhances adaptability to clinical variations over time.^3^ However, despite the growing use of upadacitinib, real‐world data regarding the timing, rationale, and outcomes of dose adjustments remain limited. In this context, a structured and evidence‐based approach to dosing transitions – either escalation or de‐escalation – could significantly improve personalized treatment planning and long‐term disease management. To address this gap, our study aimed to evaluate upadacitinib dose adjustments in adult AD patients. Conducted across six dermatology centers in Tuscany, Italy, the study included patients treated with upadacitinib from May 2022 to March 2025. Statistical analyses, including chi‐squared tests, Mann–Whitney or Student's t‐tests, and Kaplan–Meier survival analysis, were performed using R version 4.3.2 to examine associations between dose, clinical variables, and drug survival. The patients included in this manuscript provided written informed consent for the publication of their case details. The study was reviewed and approved by the Comitato Etico Regionale per la Sperimentazione Clinica della Toscana – AREA VASTA SUD EST on 16 May 2022 (protocol number 22045).

A total of 58 patients were enrolled, comprising 31 males (53.4%) and 27 females (46.6%) with a mean age of 32.3 ± 12.7 years. No patients over 65 years old were involved. Of the enrolled patients, 40/58 (69.0%) began treatment with the 15 mg dose, while 18/58 (31.0%) started at 30 mg. Baseline comparisons revealed that patients receiving the 30 mg dose had significantly higher EASI (Eczema Area and Severity Index) scores (23.33 vs. 18.08, p = 0.038), indicating more severe disease. No significant differences were observed in demographic variables, family history, disease onset, or distribution of AD phenotypes – such as classical flexural, psoriasiform, diffuse, hand eczema, or erythrodermic types – except for greater neck involvement in those starting on 15 mg (p = 0.044). During the study period, a total of 24 dose adjustments were recorded (Table 1). These were notably more frequent among patients treated with 30 mg (total 29: 18 initial prescriptions plus 11 dose escalations), with 13/29 (44.8%) requiring a dose change, compared to only 11/53 (20.8%) of those on 15 mg (total 53: 40 initial prescriptions plus 13 dose reductions). The main reasons for 30 mg dose reductions were adverse events (AEs), registered in 9/29 (31%) patients, and achievement of minimal disease activity (MDA) by 5/29 (17.2%) patients, as defined by Silverberg et al.^4^ The 9 reported AEs occurred after an average of 13.1 ± 8.5 weeks and included 4/9 acneiform reactions (44.4%), 3/9 hypercholesterolemia/hyperlipidemia (33.3%), 2/9 neutropenia/leukopenia (22.2%), and 1/9 asthenia (11.1%). Among those with AEs and dose reduction, 77.8% had already reached MDA, which was sustained in 57.1% of cases. Conversely, the sole reason for increasing the 15 mg dose was failure to achieve MDA in 11/53 cases (21.2%). These patients escalated to 30 mg after an average of 34.8 ± 26.5 weeks, achieving MDA in 40% of cases. ODS curves, excluding MDA events, are presented in Figure 1.

**TABLE 1:** Upadacitinib dose adjustments. Summary of individual dose adjustments in 20 patients. Time points (weeks) indicate when adjustments occurred. Increases from 15 mg to 30 mg were made due to inadequate disease control, while reductions from 30 mg to 15 mg were primarily driven by adverse events or achievement of minimal disease activity (MDA).

Patient	Age & Sex	Dose adjustment	Reason of adjustment	Time of adjustment (weeks)
1	34, F	15 → 30	MDA not reached	10
2	28, M	30 →15 → 30	AE: Acne (dose reduced) MDA not reached (dose increased)	16; 50
3	46, M	30 → 15	AE: Acne	12
4	19, M	15→ 30	MDA not reached	64
5	34, F	15 →30 → STOP	MDA not reached both with 15 and 30	25; 32
6	24, F	15→ 30	MDA not reached	27
7	23, M	15 → 30	MDA not reached	72
8	37, F	30 → 15	AE: acne, hyperlipidemia	12
9	60, M	30 → 15	MDA reached	17
10	30, F	30 → 15	AE: Hypercholesterolemia	13
11	28, M	30 → 15	AE: Asthenia	4
12	56, M	30 → 15	MDA reached + AE: hypercholesterolemia	10
13	20, M	15 → 30 → 15	MDA not reached (dose increased) AE: acne (dose reduced)	10; 19
14	29, F	30 → 15	MDA reached	10
15	42, F	15 → 30	MDA not reached	25
16	20, F	30 → 15 → 30	AE: leukopenia (dose reduced) MDA not reached (dose increased)	8; 103
17	27, M	30 → 15	MDA reached	13
18	16, F	15 → 30	MDA not reached	39
19	48, F	15 →30→ 15	MDA not reached (dose increased) AE: neutropenia (dose reduced)	16; 40
20	26; M	30 → 15 → STOP	MDA reached; MDA not reached and clinical decision to switch to anti‐IL	16; 29

*Abbr*.: AE, adverse event; F, female; M, male; MDA, minimal disease activity

**FIGURE 1:**
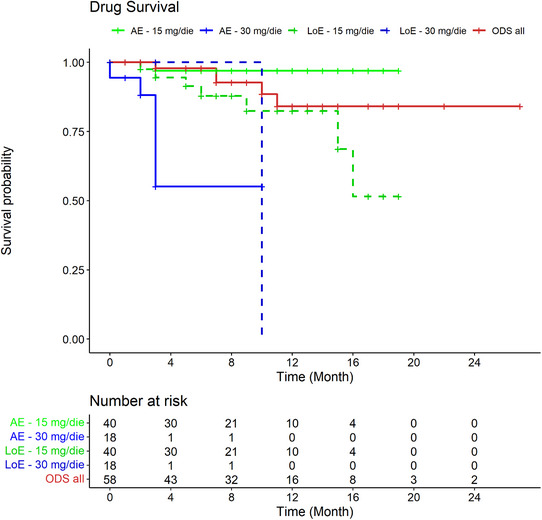
Overall Drug Survival (ODS) of upadacitinib and its different dosages. *Red line*: total cohort of atopic dermatitis patients treated with upadacitinib; *continuous green line*: drug survival of upadacitinib 15 mg/day related to adverse events; dotted green line: 15 mg/day related to loss of efficacy; continuous blue line: 30 mg/day related to adverse events; dotted blue line: 30 mg/day related to loss of efficacy. *Abbr*.: AE, adverse event; LoE, loss of efficacy; ODS, overall drug survival

This is the first real‐world study analyzing clinical factors behind upadacitinib dose changes in AD. While prior trials highlighted efficacy differences between 15 mg and 30 mg, none described the dose adjustment process.^5^ A recent review recommended maintaining patients on the lowest effective JAK inhibitor dose while allowing individualized adjustments.^6^ Our findings support this model: 15 mg was insufficient in about 20% of patients, while one in five on 30 mg could reduce to 15 mg after 13.2 weeks, maintaining MDA in 50% of cases. This timeframe is shorter than for dupilumab (52 weeks) and tralokinumab (16 weeks).^7^, ^8^ Though AEs were more frequent with 30 mg, they were generally manageable, especially acne, a common JAK‐associated side effect considered mild.^9^ Future studies should investigate predictive markers for AEs and response, as done for biologics.^10^ In conclusion, these real‐world results highlight the effectiveness, safety, and flexibility of upadacitinib, supporting its role in the personalized management of moderate‐to‐severe AD.

## CONFLICT OF INTEREST STATEMENT

A.E. has received honoraria or fees from Abbvie, Almirall, Incyte, LeoPharma, Lilly, Pfizer, and Sanofi. F.M.M. has received payments from Abbvie, Almirall, Eli Lilly, LeoPharma, Pfizer, and Sanofi. M.M. has received compensation from Abbvie and Pfizer. M.R. has received support from Abbvie, Almirall, Convatec, Eli Lilly, Janssen, LeoPharma, Novartis, Sanofi, UCB, and Urgo. V.D. has received payments from Abbvie, Almirall, Convatec, Eli Lilly, Janssen, LeoPharma, Novartis, Pfizer, Sanofi, and UCB.
